# Body Feminization Surgery 4.0: Do We Need a Safety Update?

**DOI:** 10.1007/s00266-026-06206-2

**Published:** 2026-08-24

**Authors:** Alexander Aslani, Gustavo Frontado, Alireza Arani

**Affiliations:** Department of Plastic and Aesthetic Surgery, Cirumed Clinic Marbella, Edif. Panorama, Ground Floor, Local 2, Ctra. de Cádiz, km 184, 29603 Marbella, Málaga, Spain

**Keywords:** Transgender, Body feminization surgery, Venous thromboembolism, Rib remodeling, Brazilian butt lift, Doppler ultrasound

## Abstract

**Background:**

Body feminization surgery (BFS) is an evolving component of gender-affirming care that increasingly incorporates hybrid approaches such as circumferential liposuction, gluteal augmentation, and minimally invasive rib remodeling. While these combinations yield favorable aesthetic outcomes, data regarding their thromboembolic safety profile remain limited.

**Objective:**

To evaluate early thromboembolic events following hybrid body feminization surgery and to assess the potential value of scheduled postoperative Doppler ultrasound surveillance.

**Methods:**

A retrospective review was performed of 52 transfeminine patients who underwent hybrid BFS between November 2023 and February 2025. All patients received standardized pharmacologic and mechanical thromboprophylaxis. Doppler ultrasound examinations were scheduled on postoperative days 1, 3, 7, and 10 to detect early thromboembolic events.

**Results:**

A significant improvement in waist-to-hip ratio was observed (mean reduction 32%, *p *< 0.001). Three thromboembolic events (5.7%) were identified: two asymptomatic deep vein thromboses (3.8%) detected during routine Doppler surveillance and one symptomatic pulmonary embolism (1.9%) occurring after unauthorized early long-haul travel. Doppler screening enabled detection of otherwise clinically silent events.

**Conclusions:**

Hybrid BFS may be associated with a higher-than-expected incidence of early thromboembolic events in a moderate-risk population undergoing complex, multicomponent aesthetic surgery. Scheduled Doppler ultrasound surveillance may facilitate early detection of subclinical events and warrants further evaluation in this context.

**Level of Evidence IV:**

This journal requires that authors assign a level of evidence to each article. For a full description of these Evidence-Based Medicine ratings, please refer to the Table of Contents or the online Instructions to Authors www.springer.com/00266.

## Introduction

Body feminization surgery (BFS) accounts for approximately 28.3% of all gender-affirming surgical procedures in male-to-female (MTF) transgender patients in the USA [1]. Among these, body contouring techniques—particularly liposuction and gluteal augmentation—have seen increasing demand and technical refinement [2]. This reflects not only a growing population of individuals seeking gender affirmation, but also a shift in patient expectations toward highly individualized, aesthetic-focused outcomes.

Gluteal shaping has emerged as a central component of BFS due to its pivotal role in enhancing the feminine silhouette and reinforcing gender identity [3]. Combined fat grafting and buttock implant approaches provide greater reliability in achieving volume, projection, and curvature, with outcomes that closely approximate cisgender female anatomy [4]. The primary aesthetic goal remains a waist-to-hip ratio (WHR) between 0.7 and 0.8, which has been widely accepted as an idealized feminine proportion in both clinical and anthropometric literature [4, 5].

In recent years, procedural innovation has expanded to include lower rib remodeling as an adjunct technique for waist narrowing. Traditional rib resection, while effective, remains controversial due to its invasiveness, prolonged recovery, and potential complications [6, 7]. As a result, minimally invasive alternatives—particularly the monocortical osteotomy method introduced by Kudzaev—have gained clinical interest [8]. This approach allows for targeted remodeling of ribs 11 and 12 (and, in select cases, rib 10), followed by external corset compression to stabilize the repositioned segments and achieve long-term contour enhancement (Figs. 1, 2, 3, 4, 5 and 6).

**Fig. 1:**
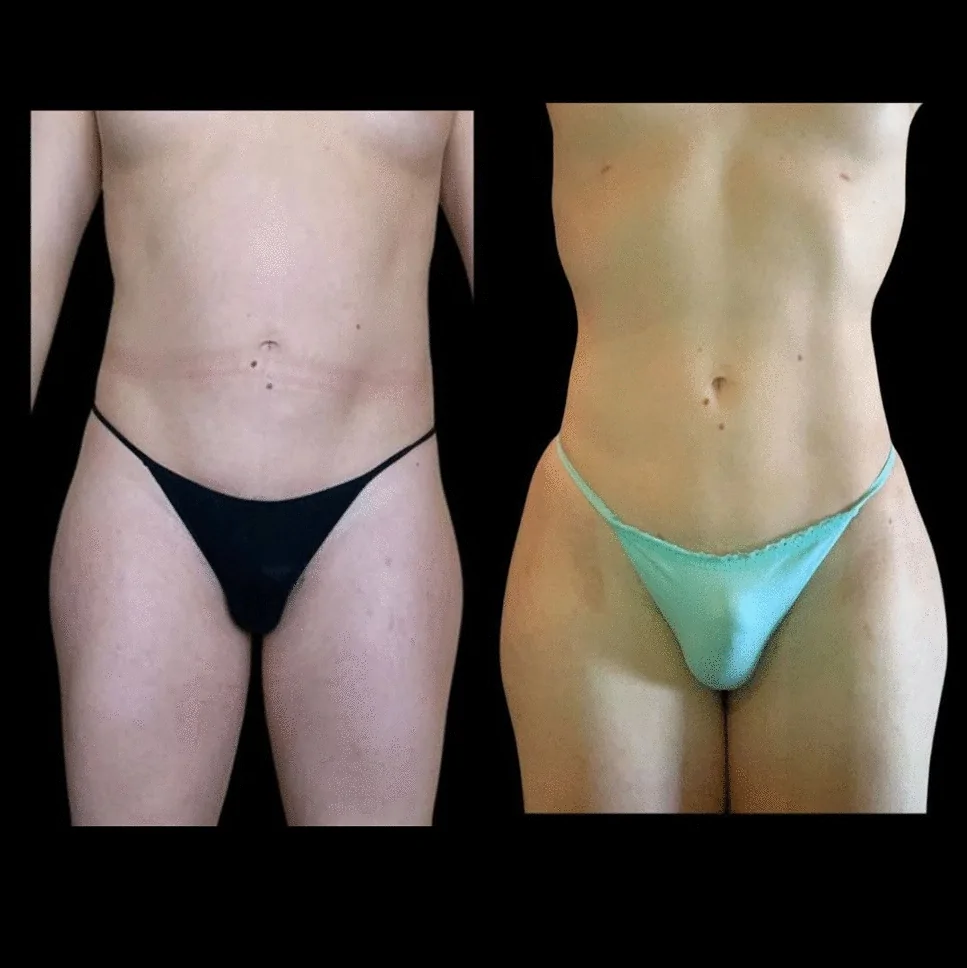
Patient A. Frontal pre- and six-month postoperative views

**Fig. 2:**
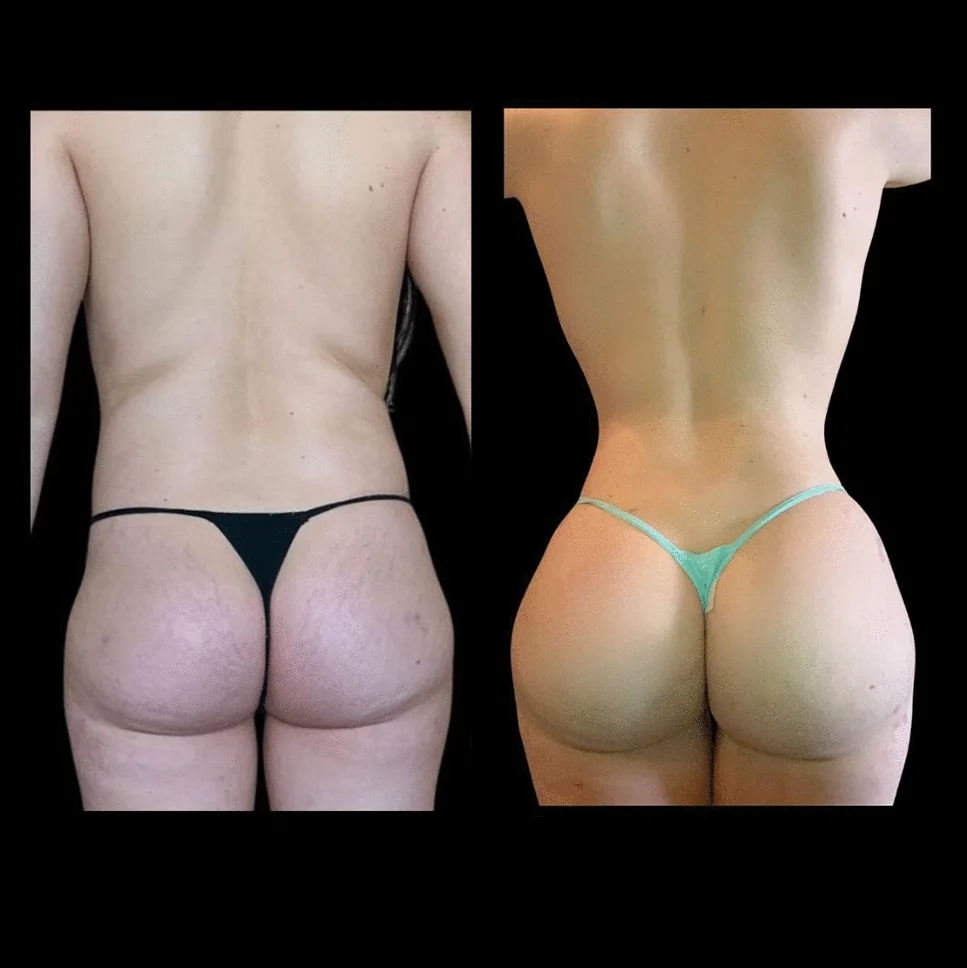
Patient A. Posterior pre- and six-month postoperative views showing waist narrowing and contour definition

**Fig. 3:**
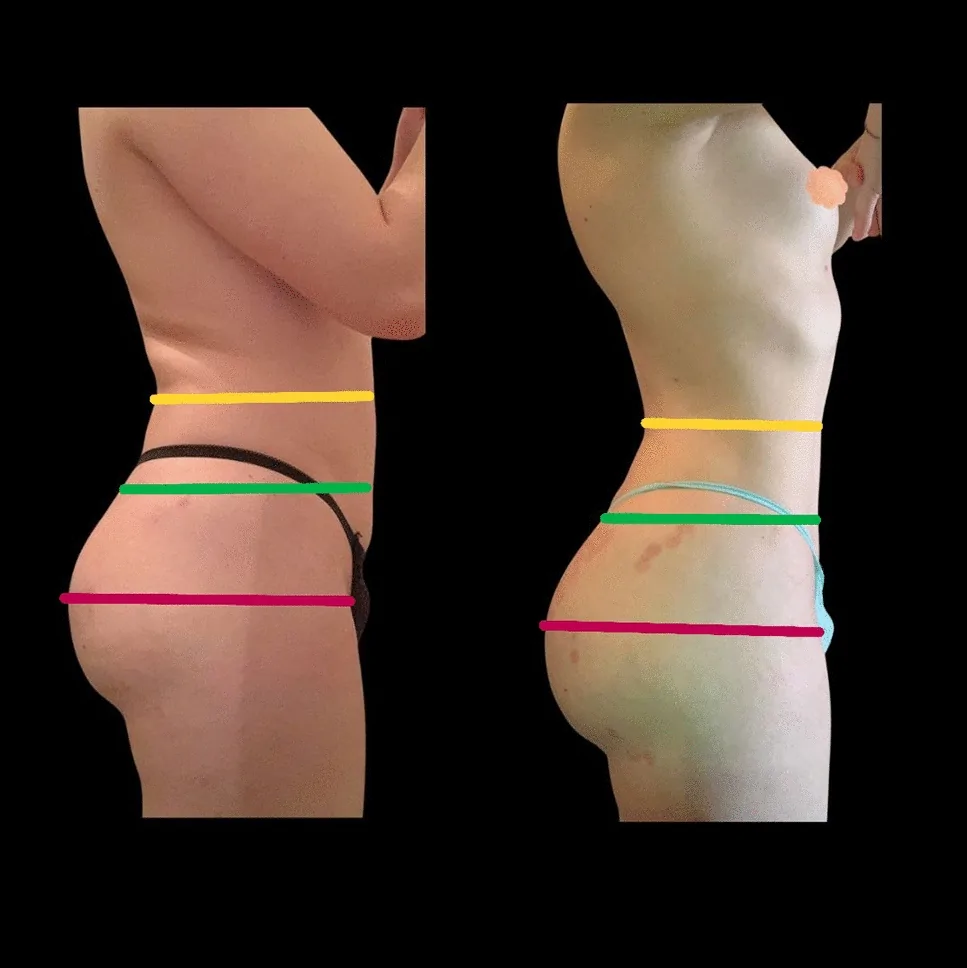
Patient A. Lateral pre- and six-month postoperative views illustrating improved waist-to-hip ratio and enhanced gluteal projection

**Fig. 4:**
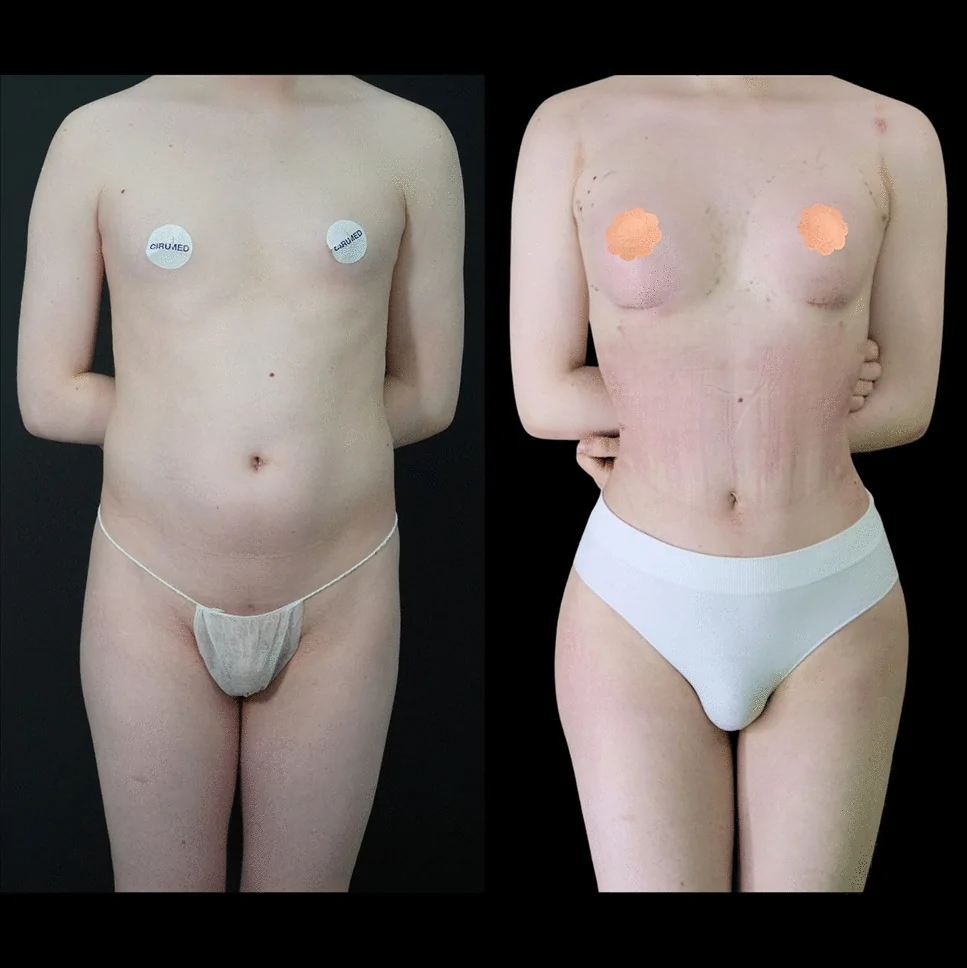
Patient B. Frontal pre- and six-month postoperative views

**Fig. 5:**
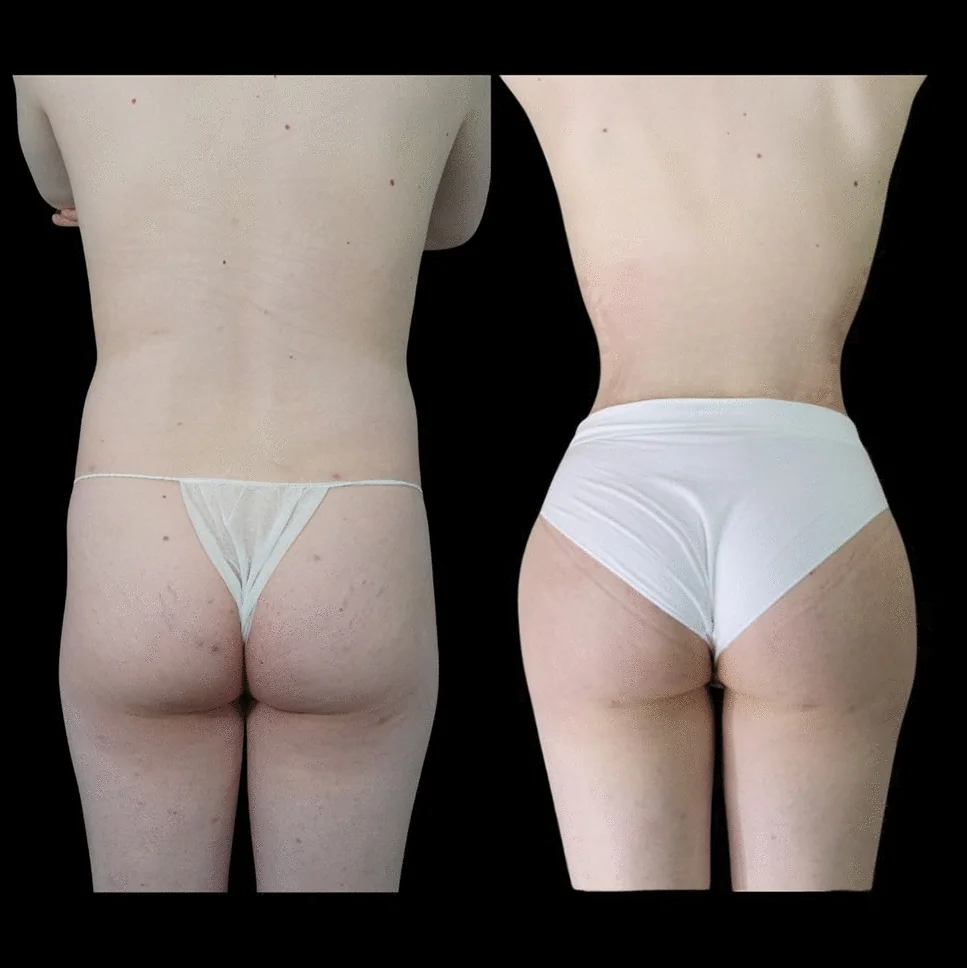
Patient B. Posterior pre- and six-month postoperative views demonstrating waist narrowing and gluteal volume enhancement

**Fig. 6:**
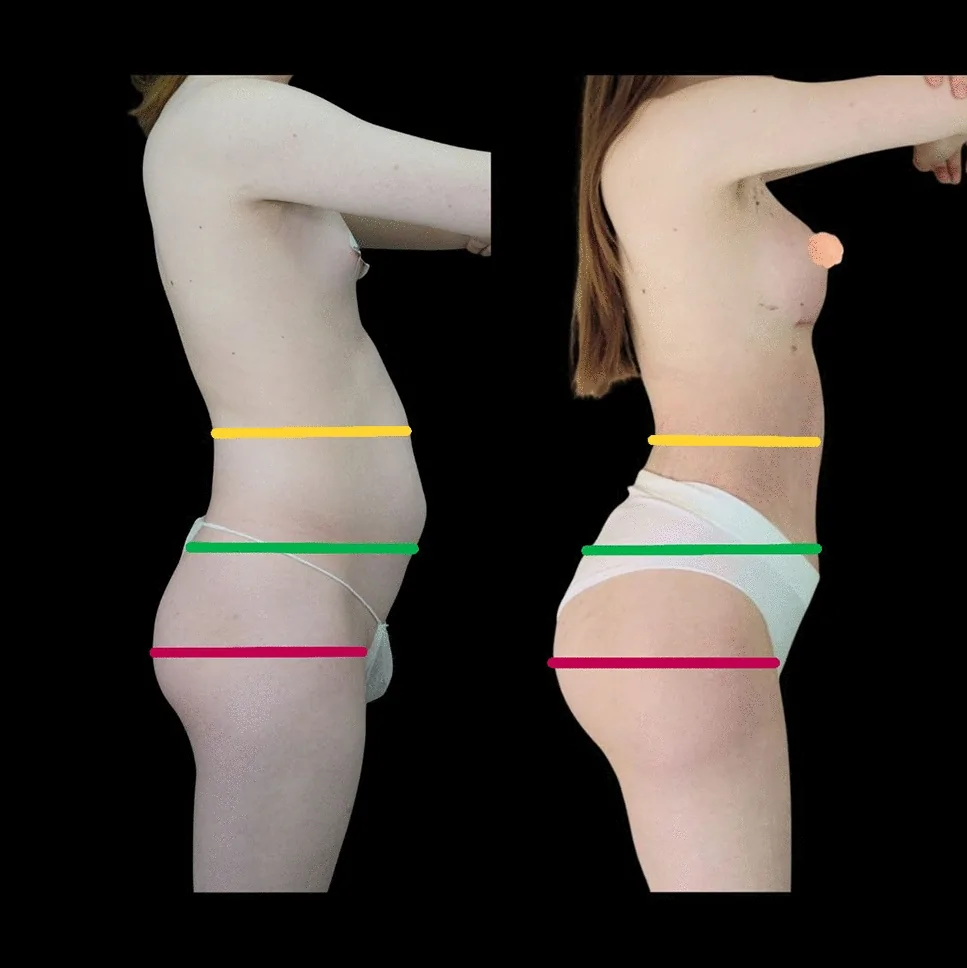
Patient B. Lateral pre- and six-month postoperative views showing improved waist-to-hip ratio and overall feminine silhouette

We have incorporated this technique into our BFS workflow with promising results, noting it to be safe, surgically efficient, moderately invasive, and highly compatible with adjunctive procedures such as liposuction, gluteal fat transfer, and implant-based augmentation. Its appeal among patients and surgeons alike has fueled a global surge in interest, making it a rapidly trending component of aesthetic gender-affirming surgery.

This study aims to evaluate the aesthetic efficacy and early safety profile of hybrid BFS—including rib remodeling—in a transfeminine population. Special emphasis is placed on thromboembolic events, given the combined effects of multizonal body contouring, operative duration, postoperative positioning, and the use of external compression garments. We also assess the potential value of scheduled Doppler ultrasound surveillance as a proactive strategy for early detection of clinically silent venous thromboembolism.

The objective of this study was to evaluate both aesthetic outcomes and early thromboembolic event rates following hybrid body feminization surgery in a transfeminine population, with particular emphasis on the role of scheduled postoperative Doppler ultrasound surveillance in detecting clinically silent venous thromboembolism.

## Materials and Methods

A retrospective review was conducted of 52 male-to-female (MTF) patients who underwent hybrid body feminization surgery (BFS) at our clinic between November 2023 and February 2025. Institutional ethical approval was obtained. Due to the retrospective design, informed consent was waived. All patients had previously consented to the use of anonymized clinical data for research purposes.

Inclusion criteria were completion of at least two years of gender transition and having undergone a hybrid surgical procedure consisting of liposculpture combined with gluteal fat grafting (BBL), with or without gluteal implants, as well as rib remodeling. Exclusion criteria included known coagulopathies, a history of thromboembolic events, active tobacco use, and preexisting respiratory disorders. All patients were confirmed non-smokers and had discontinued hormone therapy two weeks prior to surgery. The mean age was 32.1 years (range, 24–51), and the average Caprini risk score was 3.8, consistent with moderate thrombotic risk. Demographic data of the cohort are summarized in Table 1.

**Table 1:** Patient demographics and surgical characteristics

Variable	Value
Number of patients	52
Age (mean ± SD)	32.1 ± 6.7 years
Age range	24–51 years
Years since gender transition	≥ 2 years (inclusion criterion)
Hormone therapy paused pre-op	Yes (2 weeks before and after)
Active smokers	0 (all confirmed non-smokers)
Caprini score (mean ± SD)	3.8 ± 0.5
Liposuction volume (range)	2,100–4,800 mL
Gluteal fat graft volume (range)	600–1,200 mL
Gluteal implants used	18 patients (34.6%)
Implant size (mean ± SD)	340 ± 28 cc
Rib remodeling performed	52 patients (100%)
Operative time (BBL + ribs)	140 min (average)
Operative time (+ implants)	180 min (average)

Summary of baseline characteristics, procedural details, and surgical parameters in the study cohort. All patients underwent 360° liposuction, gluteal augmentation (fat grafting ± implants), and minimally invasive rib remodeling. Caprini score was calculated preoperatively to estimate thrombotic risk

Patients were instructed to pause hormone therapy two weeks before and for an additional two weeks after surgery to mitigate potential hypercoagulability.

All patients underwent 360° liposuction, gluteal fat grafting, and rib remodeling. Preoperative three-dimensional computed tomography (3D CT) scans were performed to evaluate the morphology of ribs 10 through 12, specifically assessing rib length and posterior angulation. This preoperative imaging was instrumental in customizing the osteotomy plan for each patient, allowing precise targeting of curvature and projection goals. Postoperative CT imaging was reserved for patients presenting with suboptimal outcomes or prolonged postoperative discomfort.

Liposuction was performed in both supine and prone positions following the principles of the SAFE liposuction technique, using power-assisted liposuction (PAL) device (Vibrasat® Pro, Moeller Medical GmbH). The harvested adipose volume ranged from 2,100 to 4,800 mL and was processed through gravity decantation without centrifugation. Gluteal fat grafting was performed under real-time ultrasound guidance, utilizing the expansion vibration lipofilling (EVL) technique as described by del Vecchio [9]. Reinjection was carried out under continuous ultrasound monitoring, following the technique outlined by Pazmiño [10]. Final reinjection volumes ranged from 600 to 1,200 mL, depending on patient morphology and soft tissue compliance.

Gluteal implants were placed in 18 patients, with implant volumes ranging from 275 to 390 cc (mean 340 cc), using a dual-plane pocket technique. In all cases, round, smooth silicone implants were employed. Implant selection was based on preoperative gluteal projection goals and soft tissue envelope analysis. The specifics of our dual-plane technique were previously described in detail in 2023 [11]. Rib remodeling involved a monocortical osteotomy of the targeted ribs using a piezoelectric surgical instrument (piezotome), followed by a controlled greenstick fracture confirmed intraoperatively by ultrasound. For postoperative analgesia, intercostal nerve blocks were administered using 3 mL of 0.25% bupivacaine per intercostal nerve involved.

The average operative time was 140 min for patients undergoing BBL with rib remodeling alone, and 180 min when gluteal implants were also included. Patients were maintained in a supine position for the first 24 h following surgery, then repositioned to prone for the subsequent four weeks. Rib support therapy progressed from a rigid rib-support brace during the initial five days to a structured compression corset for the remainder of the early recovery phase. Pulmonary hygiene was reinforced using incentive spirometry multiple times daily.

Thromboprophylaxis consisted of enoxaparin 40 mg initiated eight hours postoperatively and continued for 10 days, in accordance with institutional protocol for patients with a moderate Caprini risk profile undergoing extended aesthetic procedures. Mechanical compression devices were applied intraoperatively and postoperatively in all cases.

Given the multicomponent nature of the surgical procedure, operative duration, postoperative positioning requirements, and the use of external compression garments, scheduled Doppler ultrasound surveillance was implemented as an institutional early-detection strategy rather than as a universal screening recommendation. Doppler examinations were performed on postoperative days 1, 3, 7, and 10 to identify clinically silent thromboembolic events during the period of highest expected risk.

## Results

### Aesthetic Outcomes

All patients demonstrated a statistically significant improvement in waist-to-hip ratio (WHR) following hybrid body feminization surgery. The mean WHR reduction was 32% (95% CI: 28.1–35.9%; *p *< 0.001), reflecting a marked enhancement in feminine body contour consistent with the surgical objectives.

## Thromboembolic Events

Three thromboembolic events were identified in the cohort (5.7%). Two patients (3.8%) were diagnosed with asymptomatic deep vein thrombosis. One event was detected on postoperative day 7 during scheduled Doppler ultrasound surveillance. A second asymptomatic DVT was confirmed on postoperative day 8 on follow-up duplex ultrasound.

One patient (1.9%) developed a symptomatic pulmonary embolism diagnosed on postoperative day 6 after undertaking unauthorized long-haul air travel against medical advice. Due to early departure, the scheduled Doppler ultrasound examination on postoperative day 7 could not be performed. The patient subsequently informed our team of hospital admission for pulmonary embolism on postoperative day 10.

Following diagnosis, patients with deep vein thrombosis were transitioned from prophylactic anticoagulation to therapeutic management. In the case of pulmonary embolism, therapeutic anticoagulation with rivaroxaban (Xarelto®) 20 mg once daily was initiated immediately, accompanied by a 48-hour period of restricted mobilization. Anticoagulation therapy was continued for a total duration of three months. No progression of thromboembolic disease or additional thromboembolic complications were observed during follow-up. These event-specific details are summarized in Table 2.

**Table 2:** Thromboembolic events, detection, management, and clinical course

Event	Nº	%	POD	Detection	Management and outcome
Asymptomatic DVT 1	1	1.9%	7	Scheduled Doppler ultrasound	Therapeutic anticoagulation; no progression during follow-up
Asymptomatic DVT 2	1	1.9%	8	Follow-up duplex ultrasound	Therapeutic anticoagulation; no progression during follow-up
Symptomatic PE	1	1.9%	6	Hospital diagnosis after unauthorized early long-haul travel	Rivaroxaban 20 mg once daily for 3 months; no additional thromboembolic complications
Total thromboembolic events	3	5.7%	–	–	–
Events detected by routine Doppler surveillance	2 of 3	66.7% of events	7 and 8	Doppler/duplex ultrasound	Therapeutic anticoagulation; no progression during follow-up

Event-specific incidence, timing, detection method, management, and clinical course of thromboembolic events in the postoperative period. All patients underwent scheduled Doppler ultrasound surveillance on postoperative days 1, 3, 7, and 10. Two of three events were asymptomatic DVTs detected through ultrasound surveillance, supporting the potential value of early monitoring for clinically silent thromboembolic events in selected complex hybrid cases. DVT, deep vein thrombosis; PE, pulmonary embolism; POD, postoperative day

## Discussion

Our study identified a total thromboembolic event rate of 5.7%, including two asymptomatic deep vein thromboses and one symptomatic pulmonary embolism.

This finding should be interpreted with caution. Because two of the three thromboembolic events were asymptomatic DVTs detected through scheduled Doppler surveillance, the observed rate may partly reflect active detection of clinically silent events [12, 13]. In addition, the Caprini score may not fully capture all perioperative variables present in complex hybrid body contouring procedures, such as operative duration, postoperative positioning requirements, external compression garments, and early mobility limitations. Hormone therapy may represent an additional consideration in the assessment of thromboembolic risk in transfeminine patients [14]. The pulmonary embolism occurred after unauthorized early long-haul travel, representing an additional independent confounding factor. Therefore, our findings should be viewed as an early safety signal supporting careful perioperative assessment and selected postoperative surveillance, rather than as evidence that hybrid body feminization surgery is inherently unsafe.

Published literature on liposuction and subcutaneous gluteal fat grafting has addressed the safety profile and major complications associated with these procedures [15–18], suggesting that overall procedural complexity rather than any individual technique may be the primary driver of risk in hybrid cases. Rib remodeling, as performed in this cohort, should therefore be interpreted as one component within a multistep surgical approach rather than as an isolated causal factor.

The detection of asymptomatic deep vein thromboses through scheduled Doppler ultrasound surveillance underscores the potential value of early monitoring strategies in complex aesthetic procedures, particularly when traditional risk stratification tools may underestimate cumulative perioperative risk. While routine Doppler screening is not currently standard practice in aesthetic surgery, the institutional protocol applied in this study reflects a pragmatic approach to early detection of clinically silent thromboembolic events. Standard pharmacologic thromboprophylaxis with low-molecular-weight heparin provides partial protection against venous thromboembolism [19, 20]. In this context, our findings suggest that scheduled Doppler ultrasound surveillance may facilitate earlier detection of clinically silent thromboembolic events in selected patients undergoing complex hybrid body feminization surgery.

Our analysis focused on the early postoperative period, during which thromboembolic events are most likely to occur. Ongoing follow-up and future prospective studies will be required to better characterize late thromboembolic risk and to refine prophylactic and monitoring strategies in patients undergoing complex hybrid body feminization procedures.

## Limitations

This study has several notable limitations.

First, the sample size of 52 patients, while reflective of a highly specific surgical population, limits the statistical power and generalizability of our findings. Second, the absence of a control group precludes direct comparison with alternative surgical protocols or prophylactic regimens. Furthermore, the retrospective nature of the analysis introduces the possibility of selection bias, particularly given that all patients were treated at a single institution by a dedicated surgical team with a standardized technique.

We acknowledge the absence of a control group as a key limitation, which restricts direct comparison of thromboembolic outcomes with other body contouring protocols. However, our intention was not to quantify risk comparatively, but rather to provide an early signal and raise clinical awareness regarding thromboembolic vulnerability in this specific population. Future studies with prospective, controlled designs are warranted to validate these observations.

Additional confounding variables were not fully standardized across the cohort. These include variations in estrogen dosing regimens and differences in corset compression levels during postoperative recovery, all of which may influence thromboembolic risk but were not uniformly measured or controlled for in this study.

Importantly, one major thromboembolic event—the pulmonary embolism—occurred in a patient who undertook long-haul air travel within the first postoperative week, in direct violation of postoperative instructions. While clinically significant, this event is likely attributable, at least in part, to this external risk factor and should not be interpreted strictly as a surgery-related complication.

We acknowledge the lack of a control group as a limitation. Our aim was not comparative risk quantification but to raise early awareness of thromboembolic vulnerability in this specific patient group. Prospective, controlled studies are needed to confirm these findings.

Finally, Doppler ultrasound screening was performed routinely only up to postoperative day 10. Although this early surveillance window enabled detection of asymptomatic DVTs, it may have missed late-onset thromboembolic events occurring beyond that period.

## Conclusion

Hybrid body feminization surgery can achieve highly satisfactory aesthetic outcomes and represents an important and evolving component of gender-affirming care. However, the combination of multiple body contouring procedures within a single operative setting may be associated with an increased incidence of early venous thromboembolic events, even among patients classified as having a moderate baseline thrombotic risk.

While standard pharmacologic thromboprophylaxis remains essential, these findings suggest that cumulative procedural complexity and postoperative factors should be carefully considered when planning hybrid aesthetic interventions. In selected high-complexity cases, targeted postoperative surveillance strategies, such as scheduled Doppler ultrasound, may facilitate early detection of clinically silent thromboembolic events.

These results should be interpreted as an early safety signal rather than definitive evidence of causality. Further prospective, controlled studies are warranted to better define optimal risk stratification, prophylactic measures, and monitoring protocols in patients undergoing complex hybrid body feminization surgery.
